# Endogenous Causes of Obturator Nerve Entrapment: Literature Review and Proposal of a Treatment Algorithm

**DOI:** 10.3390/jcm14062068

**Published:** 2025-03-18

**Authors:** Sandra Scharfetter, Florian Wimmer, Elisabeth Russe, Karl Schwaiger, Peter Pumberger, Laurenz Weitgasser, Gottfried Schaffler, Gottfried Wechselberger

**Affiliations:** 1 Department of Plastic, Reconstructive and Aesthetic Surgery, Hospital of St. John of God, Paracelsus Medical University, Kajetanerplatz 1, 5010 Salzburg, Austria; florian.wimmer@bbsalz.at (F.W.); elisabeth.russe@bbsalz.at (E.R.); karl.schwaiger@bbsalz.at (K.S.); pumberger.peter@gmx.at (P.P.); laurenz.weitgasser@bbsalz.at (L.W.); gottfried.wechselberger@bbsalz.at (G.W.); 2 Department of Radiology and Nuclear Medicine, Hospital of St. John of God, Paracelsus Medical University, Kajetanerplatz 1, 5010 Salzburg, Austria; gottfried.schaffler@bbsalz.at

**Keywords:** obturator neuropathy, obturator nerve entrapment, nerve compression syndromes, obturator foramen, neurolysis, peripheral nerve surgery

## Abstract

**Background:** Obturator nerve entrapment can result from endogenous and exogenous causes. Due to its long course, which includes both endopelvic and exopelvic segments, the nerve is susceptible to irritation from multiple etiologies. However, as obturator nerve entrapment is relatively uncommon, a thorough understanding of endogenous factors contributing to nerve entrapment is lacking. Nevertheless, understanding the endogenous factors contributing to obturator nerve entrapment is crucial for an effective treatment approach. **Material and Methods**: We performed a systematic literature search on studies investigating the diagnostic and (surgical) therapeutic approaches to obturator neuropathy due to endogenous causes. Studies were grouped according to the etiology responsible for nerve irritation. Lastly, data were synthesized to create a clinical work-up flowchart for obturator nerve entrapment syndromes due to endogenous causes. **Results:** Data from 45 studies comprising 175 patients met our inclusion criteria. We were able to summarize these data into six broad etiologies (tumor, obturator hernia, endometriosis, cystic lesions, vascular, and idiopathic causes) responsible for nerve irritation and saw that the most important factors for therapy are the onset of the symptoms and the anatomical localization. MRI emerged as the most valuable diagnostic tool for chronic conditions, especially in identifying the precise etiology and location of nerve compression. **Conclusions:** This review offers a structured framework for diagnosing and managing obturator nerve entrapment due to endogenous causes. We propose a diagnostic and therapeutic algorithm based on the identified etiologies to facilitate clinical decision-making.

## 1. Introduction

The obturator nerve originates from the L 2–4 nerve roots and descends to the thigh, where its motor component innervates the adductor muscle compartment, which includes the external obturator, adductor longus, adductor brevis, adductor magnus, gracilis, and pectineus muscles. Its sensory component is responsible for the innervation of the medial thigh [1]. Its course can be separated into endo- and exopelvic segments, using the passage through the obturator canal as a boundary. Along its endopelvic trajectory, the nerve travels adjacent to the medial margin of the psoas muscle, located posterior to the common iliac arteries and lateral to both the internal iliac artery and ureter. Before entering the obturator canal, it passes by the internal obturator muscle. The nerve subsequently enters the medial thigh via the obturator canal, typically bifurcating into the anterior and posterior branches; however, this division may also occur intrapelvic or within the thigh. Both branches run close to the external obturator muscle as they descend. The adductor brevis muscle delineates its branches, whereas the anterior branch progresses along the anterior aspect and the posterior branch runs posteriorly. The anterior branch passes between the adductor longus and adductor brevis muscles, while the posterior division traverses the space between the adductor brevis and adductor magnus muscles. However, the obturator nerve demonstrates notable variability in its path [2,3]. In their anatomical examination study, Prudhon et al. [2] observed that the same course was never identified in two patients. Compared to other lower extremity nerves, obturator neuropathy is considered relatively uncommon [4]. However, its extended length makes the nerve prone to irritation from various causes. In addition to exogenous factors that can lead to nerve injuries or irritation, such as fractures [5], iatrogenic damage during orthopedic [6], urologic or gynecological [7] procedures, or complications during childbirth [8], endogenous factors can also lead to nerve irritation [4]. Understanding these endogenous etiologies is crucial for tailoring diagnostic approaches and treatment strategies.

Our experience with three patients suffering from obturator nerve entrapment due to three different endogenous causes—a schwannoma, a lipoma, and a ganglion cyst—highlighted a significant gap in knowledge. Despite the existing literature, there remains a lack of systematic categorization of endogenous causes of obturator nerve entrapment along with their respective diagnostic and treatment strategies. Given the role of plastic surgery in diagnosing and surgically managing nerve entrapment syndromes, we believe it is essential to integrate its expertise into a multidisciplinary approach for patients suffering from obturator nerve entrapment syndromes.

This review aims to categorize the endogenous etiologies that lead to obturator nerve entrapment, analyze their diagnostic approaches, and evaluate the effectiveness of various treatment modalities to provide a structured clinical framework.

## 2. Materials and Methods

To address the research question, we conducted a literature review, which is reported in accordance with the Preferred Reporting Items for Systematic Reviews and Meta-Analyses (PRISMA) [9] framework.

We developed a comprehensive search strategy for articles published online up to January 2025 in the PubMed database. The keywords used included (“obturator nerve” OR “n. obturator” OR “obturator neuropathy”) AND (“entrapment” OR “compression” OR “injury” OR “tumor” OR “mass” OR “schwannoma” OR “cyst” OR “lipoma” OR “neuropathy” OR “fibrosis” OR “fossa” OR “canal”) AND (“etiology” OR “cause” OR “pathophysiology” OR “pathogenesis”). The inclusion criteria for the review involved studies that reported on endogenous causes leading to obturator nerve entrapment or irritation. Research studies needed to detail their diagnostic evaluations and therapeutic approaches. Only studies involving adult patients aged 18 years and older were considered. Primary studies, including prospective and retrospective designs, as well as case reports, were accepted. Systematic and narrative reviews were analyzed for additional references but were excluded from the final data extraction. Studies that reported on multiple patients with various neuropathies were included, provided that the case of obturator neuropathy was thoroughly detailed, specifically including the causative factors and the treatment administered. Studies and individual patients referenced in this context that met these criteria were listed separately and treated as distinct cases in the results. The exclusion criteria encompassed studies in which the obturator nerve remained unaffected, studies that did not delineate the etiology of nerve entrapment, and studies focused on nerve damage attributable to exogenous factors such as injury or trauma. Furthermore, cadaver studies were also excluded.

**Figure 1:**
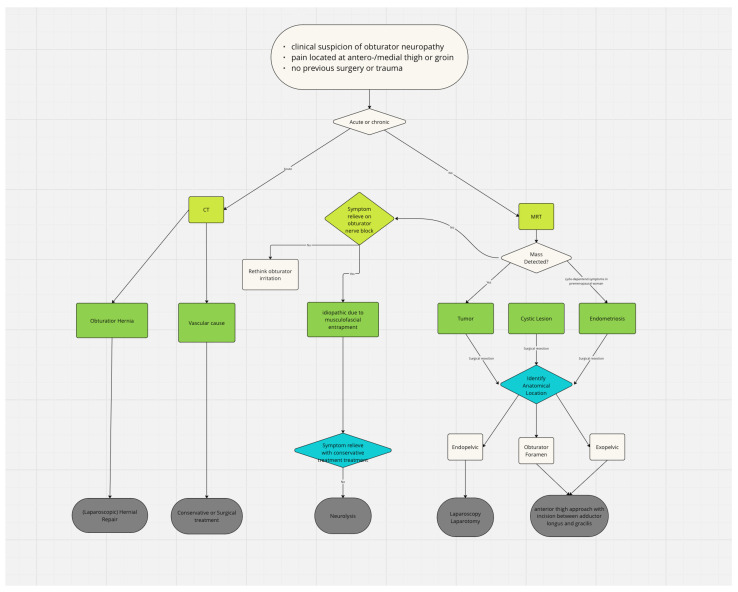
Work-Up Flow Chart—This figure illustrates the diagnostic approach for suspected obturator nerve irritation in non-traumatic, non-postoperative patients. The first step is classifying symptom onset as either acute or chronic. Acute cases should undergo CT imaging, while MRI is essential for identifying etiology and lesion location. Surgery is the most effective treatment, with the approach depending on the lesion’s location (endopelvic, obturator foramen, or exopelvic). CT or ultrasound-guided aspiration may also be considered for cystic lesions in the obturator foramen.

Initially, we screened the study titles and abstracts to exclude irrelevant articles, followed by a comprehensive review of the full texts to verify eligibility. Additionally, we examined references from the retrieved and reviewed articles to identify further pertinent studies. The extracted data can be divided into study characteristics (the authors, year of publication, and article type), patient characteristics (the number of patients, sex, and age), clinical data (the etiology of obturator nerve entrapment, and the anatomical region where the nerve was entrapped during its course—endopelvic, in the obturator foramen, or exopelvic), the diagnostic methods used, and treatment data, detailing the surgical treatment approach, treatment technique, and treatment success. After extracting the data, we categorized the studies based on the etiology of obturator nerve entrapment. To standardize the diagnostic and therapeutic approach for obturator nerve entrapment, we developed a work-up flowchart (Figure 1). This flowchart is derived from our findings and is intended to provide a structured approach for diagnosing and managing obturator nerve compression.

Given the considerable heterogeneity and low level of evidence present in the identified studies, no statistical analysis was conducted.

## 3. Results

### 3.1. Study Selection and Characteristics

**Figure 2:**
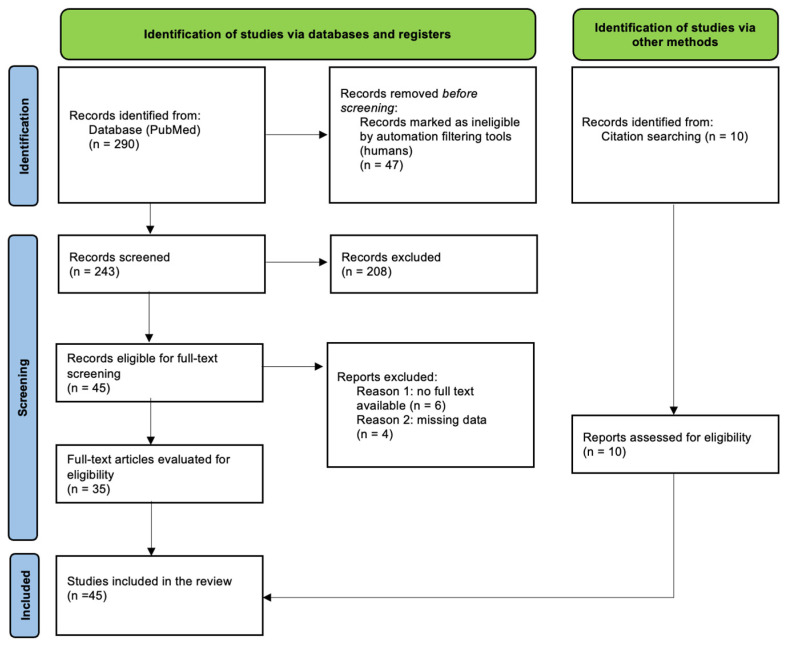
PRISMA flow chart—process of study selection.

After a comprehensive review of the literature using the established search terms, 45 studies that met all inclusion criteria were identified. The initial search yielded 290 results. Figure 2 illustrates the study selection process. After applying automatic database filters and eliminating duplicates, 243 records were deemed eligible for screening. Following a full-text review, 35 studies met the inclusion criteria. An additional 10 studies were included after screening references, resulting in a total of 45 studies included in the review.

### 3.2. Study Characteristics and Demographic Data

**Figure 3:**
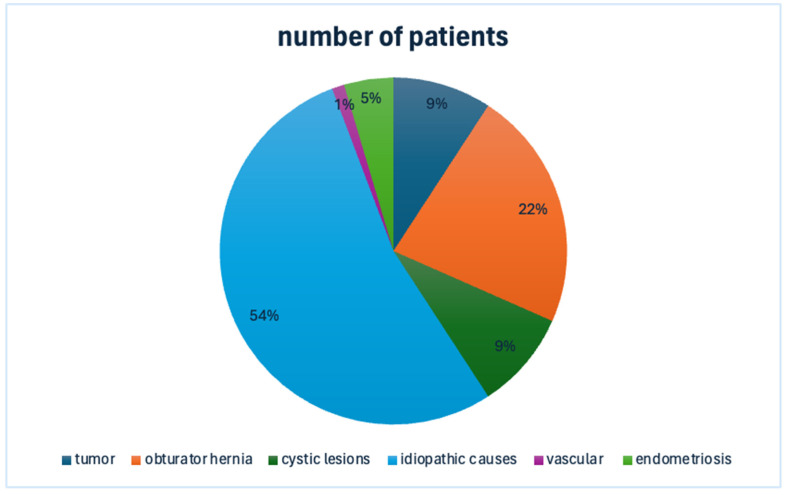
This figure illustrates the percentage distribution of the etiologies.

Out of the 45 studies included, which encompassed a total of 175 patients, the majority (37 out of 45) consisted of case reports with sample sizes ranging from 1 to 52 patients. These cases of obturator nerve entrapment were classified according to their cause, resulting in six etiological categories: tumor [10,11,12,13,14,15,16,17,18,19] (10 studies, 16 patients), obturator hernia [20,21,22,23] (four studies, 39 patients), endometriosis [24,25,26,27,28,29,30,31] (eight studies, eight patients), cystic lesions [32,33,34,35,36,37,38,39,40,41,42,43,44,45] (14 studies, 16 patients), vascular causes [46,47,48] (three studies, three patients), and idiopathic causes [49,50,51,52,53,54] (six studies, 93 patients). A high degree of heterogeneity was observed across the studies concerning diagnostic methods and treatment approaches. Figure 3 shows the percentage distribution of the causes of nerve irritation.

### 3.3. Obturator Nerve Entrapment Due to Tumors

Various types of tumors were found to cause obturator nerve entrapment, including schwannoma [10], lipoma [11,12], neurofibroma [12], parasitic leiomyoma [13], glomus tumor [14], lipomatosis of the obturator nerve [15], cancer [16,18] (including bladder transitional cell carcinoma, pelvic papillary adenocarcinoma, carcinoma of unknown primary, and non-Hodgkin lymphoma), and neurinoma [17]. Additionally, granuloma [19], although not a tumor but an inflammatory lesion, was included in this category for simplification. Tumors were located in the pelvis in 13 cases [10,12,13,15,16,17,18,19], entirely exopelvic in two [14,16], and one (a glomus tumor) was located between the adductor muscles [14]. One study reported a case where the tumor was located both endo- and exopelvic [11]. This tumor was a dumbbell-shaped lipoma extending from between the adductor magnus and brevis muscles through the obturator foramen up to the pelvis [11]. Diagnostic confirmation was primarily achieved using computed tomography (CT), magnetic resonance imaging (MRI), and ultrasound. No preoperative biopsies were performed in any of the reported cases. Surgical resection was the primary treatment in all cases except for one, in which the lipomatosis of the obturator nerve resolved with conservative management, including the application of steroids [15]. Most tumors were resected through an abdominal approach, using either laparotomy [12,16,19] or laparoscopy (with or without robotic assistance) [10,11,13]. Masses located between the adductor muscles were resected using an anterior surgical approach [11,14]. In two studies, the surgical approach was not further specified [17,18]. Treatment success was reported in all cases except for three involving cancer patients. A comprehensive summary of all included studies is presented in Table S1.

#### 3.3.1. Clinical Example 1—Schwannoma

**Figure 4:**
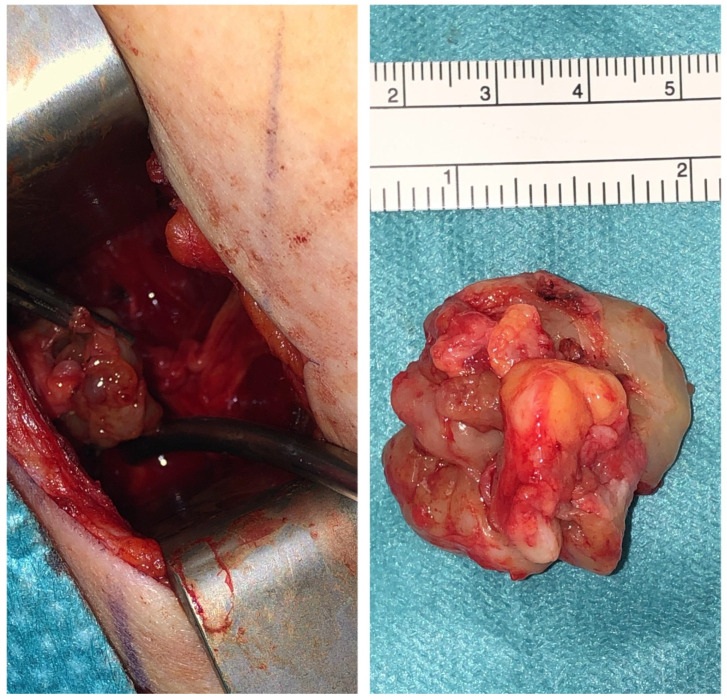
The figure shown depicts the intraoperative findings of the patient. In the left photograph, the Schwannoma is visible before resection. The mass can be seen after removal in the right photograph, measuring approximately 3.7 × 2.5 cm.

We present the case of a 26-year-old woman experiencing sensory deficits and pain in the inner thigh region with no signs of motor weakness. Magnetic resonance imaging (MRI) with contrast enhancement and ultrasound suggested a schwannoma measuring 3.7 × 2.5 cm located in the left obturator foramen, prompting surgical intervention. An incision was made between the adductor longus and gracilis muscles to access the obturator nerve. The dissection was extended proximally toward the obturator foramen, where the lesion was identified. Macroscopically, it was consistent with a schwannoma. We successfully removed the tumor while preserving the nerve (Figure 4). The surgery was performed under general anesthesia and loop magnification. Histopathological analysis confirmed the diagnosis of a schwannoma. Postoperatively, the patient is symptom-free without any sensory or motor deficits.

#### 3.3.2. Clinical Example 2—Lipoma

A 68-year-old woman presented with tugging pain in the suprapubic region. During a urological workup, an incidental finding on MRI revealed an intramuscular, contrast-enhancing lipoma measuring approximately 10 cm within the left adductor magnus muscle. The tumor extended cranially to the obturator foramen and into the pelvis, compressing the obturator nerve. Surgical resection was indicated as we suggested that the lipoma was the probable cause of the patient’s pelvic pain. The surgical approach adhered to the technique outlined in Clinical Example 1, performed under general anesthesia with loop magnification, involving an incision made between the adductor longus and gracilis muscles. The lipoma was successfully resected from the thigh to the obturator foramen. Postoperatively, the obturator nerve remained intact; however, the patient’s symptoms persisted, still without any known cause.

### 3.4. Obturator Nerve Entrapment Due to Obturator Hernia

Four studies (two case reports and two retrospective chart reviews) reported obturator hernia as a cause of nerve entrapment [20,21,22,23]. Nearly all patients (97%) were female, and nerve compression consistently occurred in the obturator canal. CT [20,22] was the most commonly utilized diagnostic modality, followed by MRI [21]. Only one study employed EMG to confirm obturator mononeuropathy [21]. In all studies except one, laparoscopic hernia repair was the preferred treatment, while conservative management was utilized in that particular case. The conservative treatment strategy was not further specified [21]. Treatment success was reported across all studies. A detailed summary of all included studies is presented in Table S2.

### 3.5. Obturator Nerve Entrapment Due to Endometriosis

Eight case reports identified endometriosis as a potential cause of obturator nerve irritation. The most frequently noted symptom was localized pain in the groin and inner thigh. In three cases, symptoms were cycle-dependent [24,30,31]. MRI was the preferred diagnostic method in 75% of cases [25,26,27,29,30,31], while the diagnosis was based solely on clinical findings in 12.5% [24]. In 12.5% of cases, the diagnostic approach used was not specified. The cause of entrapment was endopelvic in six patients [24,25,26,27,28,29], exopelvic in one [31], and both endo- and exopelvic in one [30]. In that case, endometriosis was found along the entire course of the nerve [30]. All patients underwent surgical resection, with a preoperative biopsy performed in one case [30]. Laparoscopic resection was performed in all cases where the mass was situated in the pelvis. Resection was performed using an anterior thigh approach in one case, where the endometriosis was located intramuscularly between the adductor magnus and gracilis muscles [31].

Table S3 provides a summary of the studies mentioned above.

### 3.6. Obturator Nerve Entrapment Due to Cystic Lesions

Cystic lesions included ganglion cysts [32,36,37,39,42,43,44,45], acetabular paralabral cysts [33,38,40], synovial cysts [35], and mucoid pseudocysts [41]. MRI was the diagnostic modality of choice in all cases. Cysts were located in the obturator foramen in six patients [32,34,39,42,43,44], entirely endopelvic in five patients [33,34,38,41,45], exopelvic in three [35,37,40], and both endo- and exopelvic in one case, described as an intraneural ganglion cyst [36]. A biopsy was performed in one study [39]. The anatomical location of the cyst did not determine the treatment approach. Treatment strategies included aspiration in four patients (guided by CT [32,37] or ultrasound [38,43]), arthroscopy [35,45] in two, conservative management in one [40], laparoscopic resection in one [39], open abdominal incision in four [34,41,42,44], and a transverse groin crease incision in one [33]. Treatment success was reported in all but one [36] study. The studies included in this category are summarized in Table S4.

#### Clinical Example 3—Ganglion Cyst

**Figure 5:**
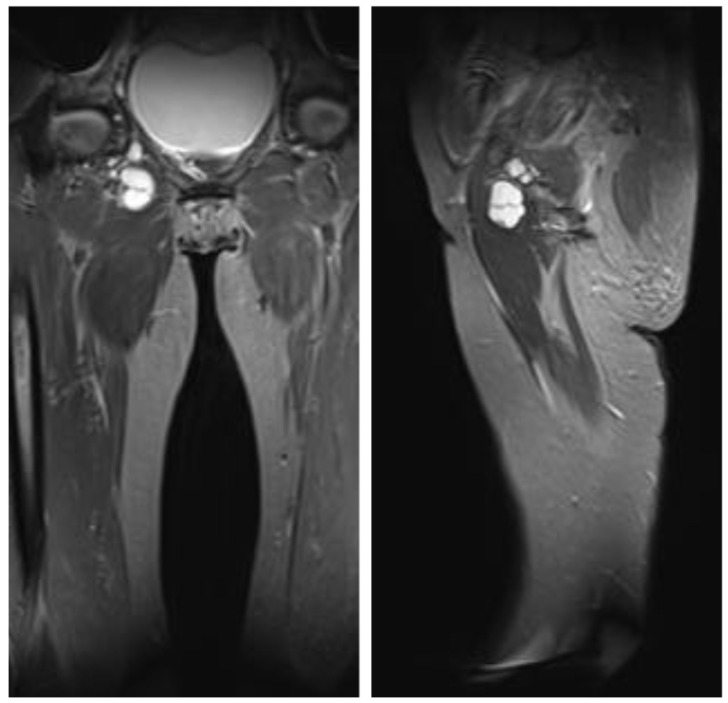
This figure illustrates the preoperative MRI T1-weighted findings with contrast enhancement of the patients (coronal and axial views), showing two cystic multiseptate lesions along the course of the obturator nerve. The larger lesion (2.4 × 2.3 × 3.0 cm) is located in the adductor magnus muscle, possibly contacting the inferior edge of the mildly arthritic right hip joint. The smaller lesion (2.0 × 1.0 × 1.6 cm) is situated more cranially in the obturator foramen.

A 50-year-old woman presented to our Department of Plastic, Reconstructive, and Aesthetic Surgery with the suspected diagnosis of an intraneural ganglion irritating the obturator nerve. The patient experienced pain in both the medial and lateral regions of the thigh. An MRI with contrast enhancement two showed cystic multiseptate lesions following the course of the obturator nerve: one located in the adductor magnus muscle (2.4 × 2.3 × 3.0 cm) with a connection to the hip joint, and another in the obturator foramen (2.0 × 1.0 × 1.6 cm) (Figure 5).

**Figure 6:**
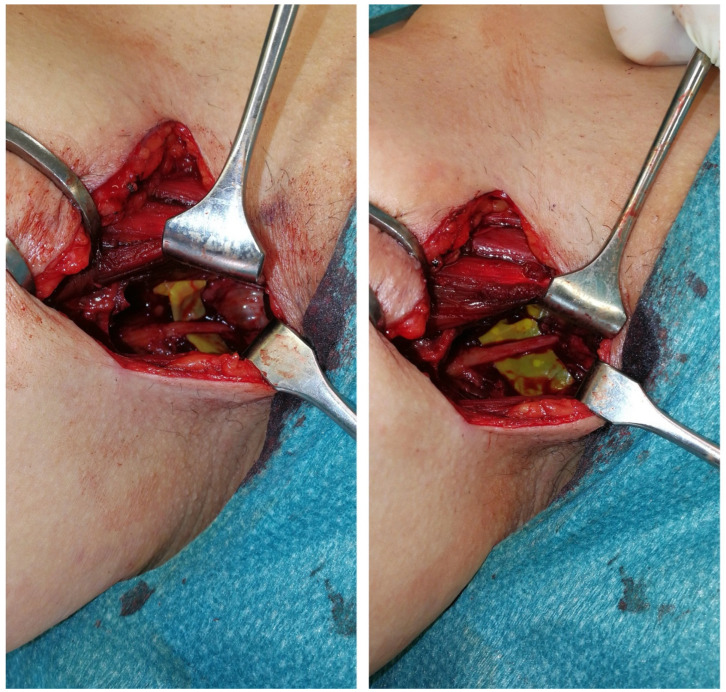
This image illustrates the intraoperative findings of the patients. In the left photo, the ganglion, measuring approximately 2 × 3 cm, can be seen in situ. In the right photo, the obturator nerve and its continuity can be appreciated after the cyst was removed.

With this finding, resection was recommended. We performed the surgical resection under loop magnification, starting with an incision between the tendon of the adductor longus and the gracilis tendon while the patient was under general anesthesia. After exposing the obturator nerve, we proceeded with further dissection proximally to the obturator foramen, where we located a ganglion measuring 2 × 3 cm. The cyst was opened, and the capsular of the cyst was followed, likely originating from the hip joint. Intraoperative stimulation of the obturator nerve confirmed consistency but with a weakened signal (Figure 6). Notably, postoperatively, the patient did not experience any deficiencies, and six years after surgery, the patient remains free of symptoms.

### 3.7. Obturator Nerve Entrapment Due to Vascular Causes

Vascular causes included hypogastric artery aneurysm [46] and compression due to retroperitoneal hemorrhage [47,48]. CT was the diagnostic modality of choice [46,48]. One patient with obturator mononeuropathy resulting from an abdominal aortic rupture died before further diagnostics could be performed [47]. The hypogastric aneurysm was treated with ligation, and the retroperitoneal hematoma was managed conservatively. Both patients experienced symptom relief. Studies in this category are listed in Table S5.

### 3.8. Obturator Nerve Entrapment Due to Idiopathic Causes

This group consisted of 93 patients exhibiting typical symptoms of obturator nerve irritation with no identifiable mass compressing the nerve [49,50,51,52,53,54]. Muscular compression or the obturator membrane due to an idiopathic cause is thought to be responsible for nerve irritation in most patients [49,50,53,54]. The patients identified with muscular entrapment were exclusively athletes, with the entrapment located in the thigh. Neurolysis was performed through an anterior surgical approach in all cases [49,50,54]. The patients without athletic backgrounds underwent laparoscopic neurolysis in the area of the obturator membrane [51,52,53]. Anesthetic nerve blocks were the most effective diagnostic tool, and EMG confirmed obturator mononeuropathy in approximately 91% of cases, all of whom were athletes. The studies categorized under that designation are presented in Table S6.

## 4. Discussion

In this study, we identified endogenous causes of obturator nerve entrapment, highlighting their potential impact on diagnosis and treatment planning. Our findings systematically summarize the current literature, offering insights into how obturator neuropathy has been previously addressed. Additionally, we present three cases of obturator nerve irritation caused by different underlying factors. Consistent with earlier studies [4], we found that obturator neuropathy is a relatively rare condition. While several reviews have addressed neuropathies of the lower extremity, including obturator neuropathy, these are primarily narrative reviews [55,56,57]. Our study expands on existing knowledge by systematically extracting data categorizing obturator nerve irritation based on its etiology and providing a proposal for a treatment approach (Figure 1).

We focused exclusively on endogenous etiologies, excluding patients with recent trauma, injury, or surgery, as their diagnostic pathway differs significantly. Most of the reviewed literature comprised case reports with varied symptom descriptions. Reported symptoms ranged from generalized leg pain to localized anteromedial thigh and groin pain. In our opinion, the most indicative symptoms of obturator nerve irritation are groin pain with or without radiation into the thigh or solely medial thigh pain, which aligns with the nerve’s sensory innervation. The exception is obturator hernia, where bowel symptoms frequently co-occur. We believe that differentiating between acute and chronic symptom onset is crucial, as the diagnostic approach varies accordingly. We found that acute obturator nerve irritation was primarily caused by obturator hernia and vascular causes such as retroperitoneal hemorrhage, with CT imaging being the preferred diagnostic modality in these cases.

We observed that most cases of idiopathic obturator nerve irritation are reported in athletes. This patient group showed symptom exacerbation during exercise, which can thus be regarded as the most significant clinical indicator. Bradshaw et al. detailed this etiology, recommending electrophysiologic studies and obturator nerve block with local anesthetic as effective diagnostic tools for idiopathic nerve issues irritation [50]. For the majority of other etiologies, we observed that electromyography (EMG) was either not used or deemed diagnostically inconclusive. For example, in cases of glomus tumors in the thigh [14] and endometriosis involving the obturator nerve, EMG results were negative for obturator mononeuropathy [29,30], despite clinical and radiological evidence of obturator nerve irritation. Based on these findings, we do not consider EMG essential for standard diagnostic evaluations, except in cases where there is no clearly identifiable mass that could be causing nerve irritation.

CT is preferred for patients with suspected obturator hernia or vascular causes responsible for obturator nerve entrapment [58]. For all other cases, we consider MRI the most essential diagnostic tool. Beyond aiding in preoperative suspicion, MRI precisely identifies the site of nerve compression, enabling more effective patient triage and treatment planning. Ultrasound serves as a valuable initial diagnostic modality, being readily accessible and providing preliminary insights into etiology, especially in consideration of recent technological advancements in high-resolution ultrasound (HRUS). This technology has demonstrated its capability to accurately identify the site of nerve irritation that correlates with intraoperative observations [59]. Although HRUS and other even more advanced imaging technologies, like magnetic resonance microscopy, can visualize affected nerve fascicles and affected nerve volume [60], we suggest that a standard MRI is adequate for cases of obturator nerve irritation related to endogenous factors. It is crucial to emphasize that the nerve is typically irritated rather than completely disrupted. In cases of nerve irritation caused by endogenous factors, advanced technologies may play a secondary role for the patient cohort under investigation, as supported by the reviewed literature. It is essential to note that none of the examined nerves seemed to exhibit loss of continuity. Furthermore, ultrasound may not provide sufficient information in cases of endopelvic nerve irritation.

Given the relatively long course of the obturator nerve, multiple potential entrapment sites can arise along both its endopelvic and exopelvic pathways. In the presence of space-occupying pathologies, nerve irritation is almost inevitable. Key entrapment sites include the obturator foramen and the intermuscular space between the adductor muscles, among others. Anatomical studies have identified several factors that may contribute to idiopathic obturator nerve entrapment, although opinions vary regarding the most critical sites [2,50]. For these cases, Bradshaw et al. recommend decompression at the obturator foramen, near the medial circumflex artery, and at the level of the adductor brevis muscle fascia [50]. Another study highlights additional potential entrapment sites, including the nerve’s passage through the obturator muscle, its course between the fascia separating the adductor brevis and longus muscles, and its close proximity to the medial branch of the circumflex artery [2].

The treatment approach varies based on the location of the pathology, whether endopelvic, within the obturator foramen, or exopelvic. Our review of the literature shows that masses located entirely within the pelvis are typically approached through an abdominal method, utilizing either laparoscopy or laparotomy, based on the underlying etiology. In cases of nerve irritation in the thigh, decompression is usually carried out through an anterior thigh approach.

For patients with irritation confined to the obturator foramen, treatment strategies vary. All our patients, one who had a lipoma, one who had a schwannoma, and one who had a ganglion cyst, were successfully treated using a technique that involved an incision between the adductor thigh muscles. In contrast, Campeas et al. [42] and Munugani et al. [44] reported cases of ganglion cysts within the obturator foramen that were resected through a lateral extraperitoneal approach. Schwabegger et al. [39] successfully removed a ganglion cyst laparoscopically. In addition to surgical resection, cysts were also treated with CT- or ultrasound-guided aspiration with favorable outcomes [32,37,38,43]. Based on these findings, we believe that the treatment approach depends on where the patient initially seeks medical attention and the surgeon’s expertise.

Furthermore, we saw that preoperative biopsy played an inferior role in managing obturator nerve entrapment. Of all the cases analyzed, only two masses were biopsied [30,39], including a case of endometriosis affecting the entire course of the nerve [30]. Our observation indicates that biopsy results might not significantly alter treatment pathways for most patients.

Based on our findings, we propose a standardized diagnostic and therapeutic approach for obturator nerve entrapment, summarized in the work-up flowchart (Figure 1). This algorithm integrates clinical assessment, imaging, and potential treatment strategies tailored to different endogenous etiologies. This structured approach can help clinicians optimize the management of obturator neuropathy.

Given the complexity of obturator nerve entrapment and its varied etiologies, we believe a multidisciplinary approach involving radiologists and, depending on the location of the entrapment, plastic surgeons, orthopedic surgeons, general surgeons, vascular surgeons, and gynecologists, is crucial for enhancing diagnostic accuracy and optimizing treatment strategies. We consider a patient-adapted approach based on factors such as symptom duration, etiology, and anatomical involvement, as illustrated in Figure 1, to be of utmost importance for achieving the most effective treatment success.

We observed that invasive treatment approaches were the preferred choice in most cases. This contrasts with the findings of Sorenson et al. in their review of obturator neuropathy, where conservative treatment was often sufficient to alleviate symptoms in patients with acute obturator neuropathy. [61]. However, it is important to note that most patients in their study suffered from obturator neuropathy due to surgical complications or trauma. Given this distinction, we believe conservative treatment has a limited role to play in managing endogenous causes of obturator irritation. However, it may be considered based on the severity of symptoms, the underlying etiology, and the patient’s overall condition. Notably, cases of retroperitoneal hemorrhage, obturator hernia, lipomatosis of the nerve, and acetabular paralabral cysts have demonstrated symptom resolution through conservative management with, for example, steroids [15,21,40,48].

Obturator neuropathy can sometimes manifest in unusual ways, with symptoms limited to the groin or pelvic area, which may cause delays in diagnosis. It is important to be aware that symptoms of obturator neuropathy can be restricted to these locations. Furthermore, in Clinical Example 2, a lipoma situated between the adductor muscles highlights the importance of considering other conditions causative for the patient’s symptoms. In our instance, the lipoma was found incidentally during an MRI aimed at discovering the cause of the patient’s lower abdominal discomfort. Despite successfully removing the mass, the patient’s symptoms persisted, necessitating further urological evaluations. However, the primary cause of symptoms remained unidentified.

## 5. Conclusions

This review provides a structured framework for diagnosing and managing obturator nerve entrapment resulting from endogenous causes. Given the diverse causes of obturator neuropathy entrapment syndromes, a multidisciplinary approach is essential for optimizing patient outcomes. The site of entrapment, whether endopelvic in the obturator foramen or exopelvic, is a significant factor in determining the appropriate treatment approach. To enhance clinical decision-making, we propose a diagnostic and therapeutic algorithm.

## Data Availability

The complete data of this study are available on request from the corresponding author, as presenting the complete data would have extended the scope of the study.

## Supplementary Materials

The following supporting information can be downloaded at: https://www.mdpi.com/article/10.3390/jcm14062068/s1, Table S1: An overview of the studies included in the “tumor” group is presented. Table S2: An overview of the studies included in the “obturator” group is presented. Table S3: An overview of the studies included in the “endometriosis” group is presented. Table S4: An overview of the studies included in the “vascular” group is presented. Table S5: An overview of the studies included in the “cyst” group is presented. Table S6: An overview of the studies included in the “idiopathic” group is presented.
