# Supplementary material for: Endogenous Causes of Obturator Nerve Entrapment: Literature Review and Proposal of a Treatment Algorithm
Source: J Clin Med. 2025 Mar 18;14(6):2068. doi: 10.3390/jcm14062068 (PMC11943024; doi:10.3390/jcm14062068)
Supplement: Supplementary file 1 [file jcm-14-02068-s001.zip › jcm-3494842-supplementary.pdf]

Authors	Year	Study Design	N	Population	sex	Described cause of nerve irritation	leading symptom	motor weakness as leading symptom	described anatomic location of irritation	location of irritation according to the pelvis	Diagnostic modality	Biopsy (yes/ no)	Mononeuropathy on EMG	Treatment approach	Treatment type	Treatment success
**Bulisani, B. M. et al.**	2024	Case Report	1	51-year-old female	f	Schwannoma	left pelvic discomfort	no	extraperitoneal located medially and inferiorly to the left external iliac vessels	endopelvic	CT	no	no	laparoscopy	resection	yes
**Kimura, K. et al.**	2020	Case Report	1	64-year old male	m	Lipoma	numbness (not further specified) increased while walking	no	between adductor magnus and brevis extending through obturator foramen into right pelvis	endo- and exopelvic	MRI	no	n/a	Open surgical using an anterior approach from the thigh and laparoscopy	resection	yes
**Kitagawa R. et al.**	2009	Retrospective Study	1	75-year-old female (neurofibroma)	f	Neurofibroma	pelvic and groin pain	no	intrapelvic (not further specified) arising from right obturator nerve	endopevic	Not furterh specified	no	n/a	laparotomy	resection	yes
**Kitagawa R. et al.**	2009	Retrospective Study	1	55-year-old female (fibrous lipoma)	f	Lipoma	inner thigh pain	yes	left obturator fossa	endopelvic	Not furterh specified	no	n/a	laparotomy	resection	yes
**Menderes G. et al**	2018	Case Report	1	48-year old female	f	Parasitic leiomyoma	back pain radiating to groin and inner thigh	no	obturator fossa	endopelvic	MRI	no	n/a	robotic-assisted laparoscopy	resection	yes
**Muller, A.**	1997	Case Report	1	62-year-old male	m	Glomus tumor	inner thigh pain	yes	intramuscular in the lower	exopelvic	ultrasound	no	no	Open surgical using an anterior ap-	resection	yes

									inner thigh					proach from the thigh		
**Nardone R. et al.**	2008	Case Report	1	61-year-old-female	f	Lipomatosis of obturator nerve intraneural tumor	groin pain radiating towards anterior and inner thigh	no	from bladder extending into obturator foramen	endopelvic	MRI	no	yes	conservative	conservative	yes
**Rogers L. R. et al.**	1993	Clinical Case Series	6	adult population (3 male and 3 female age between 52 and 81)	mixed	Cancer (Bladder TCC, Pelvic papillary adenocarcinoma, CUP, non-Hodgkin lymphona)	groin pain	yes	1: anterior lower pelvis; 2: posterolateral midpelvis anterior lower pelvis and extrinsic to bony pelvis in external obturator and pectineus muscle; 3: posterolateral midpelvis in psoas and iliacus muscle; 4: posterolateral midpelvis region of psoas and iliacus muscle; 5: posterolateral upper, mid, and lower pelvis in region of psoas	endopelvic (5/6), exopelvic (1/5)	CT	no	yes (5/6)	laparotomy	resection	yes (3/6)

									and iliacus muscle; 6: posterolateral upper, mid and lower pelvis region of psoas and iliacus muscle;							
**Stoltze D. et al**	1982	Case Report	1	39-year old female	f	Neurinoma	knee pain	no	psoas muscle	endopelvic	CT	no	n/a	not further specified	resection	yes
**Turkoglu R et al.**	2016	Case Report	1	35-year-old female	f	Adenocarcinoma of unknown primary origin	groin and thigh pain	no	lateral from left internal iliac artery	endopelvic	MRI	no	yes	not further specified	resection	yes
**Yamashita K. et al.**	2004	Case Report	1	61-year-old-female	f	Granuloma (in response to silk suture used in previous operation)	abdominal and groin pain	no	between the external and the internal iliac vessels, with its center 4 cm cephalad from the obturator foramen	endopelvic	CT	no	n/a	laparotomy	resection	yes

Table S1: An overview of the studies included in the “tumor” group is presented. The location of obturator nerve irritation is illustrated in detail and categorized based on its relationship to the pelvis. (CT: computed tomography; MRI: magnetic resonance imaging; N: number of patients; f: female; m: male; n/a: not applicable)

Authors	Year	Study Design	N	Population	sex	Descripted cause of nerve irritation	leading symptom	motor weakness as leading symptom	descripted anatomic location of irritation	location of irritation according to the pelvis	Diagnostic modality	Biopsy (yes/ no)	Mononeuropathy on EMG	Treatment approach	Treatment type	Treatment success
**De Clercq, L; et al. 2010**	2010	Case Report	1	91-year-old-female	f	Obturator Hernia	leg pain radiating upward to thigh and groin	no	obturator canal	obturator foramen	CT	no	n/a	laparoscopy	hernial repair	yes
**Mondelli, M. et al.**	2002	Case Report	1	73-year old female	f	Obturator Hernia	groin pain radiating to buttock and inner thigh	no	entrance of obturator foramen	endopelvic	MRI	no	yes	conservative	conservative	yes
**Nasir B. et al.**	2012	Retrospective Study	30	patients with obturator hernia repair (median age 82, 97% women)	mixed	Obturator Hernia	bowel obstruction (63%), abdominal/groin pain (57%), and a palpable lump (10%), positive Howship-Romber sign (11%)	no	compressiom in the canal	obturator foramen	CT	no	n/a	laparoscopy	hernial repair	yes
**Perry, C. P. and Hantes, J. M.**	2005	Retrospective Study	7	female patients with chronic pelvic pain	f	Obturator Hernia	pelvic pain	no	obturator canal	obturator foramen	not further specified	no	n/a	laparoscopy	hernial repair	yes

Table S2: An overview of the studies included in the “obturator” group is presented. The location of obturator nerve irritation is illustrated in detail and categorized based on its relationship to the pelvis. (CT: computed tomography; MRI: magnetic resonance imaging; N: number of patients; f: female; m: male; n/a: not applicable)

Authors	Year	Study Design	N	Population	sex	Described cause of nerve irritation	leading symptom	Cycle-dependent symptoms	motor weakness as leading symptom	described anatomic location of irritation	location of irritation according to the pelvic	Diagnostic modality	Biopsy (yes/ no)	Mononeuropathy on EMG	Treatment approach	Treatment type	Treatment success
**Carranco, R. C. et al.**	2021	Case Report	1	31-year-old female	f	endometriosis	cycle-dependent pain while walking	no	yes	ovarian fossa, with involvement in the area between the umbilical ligament and the uterine artery	endopelvic	clinical	no	not further specified	laparoscopy	resection	yes
**Ekpo G. et al.**	2007	Case Report	1	33-year-old female	f	endometriosis	lower extremitiy pain (not further specified)	yes	not further specified	retroperitoneally near obturator fossa encasing the obturator nerve	endopelvic	MRI	no	no	laparoscopy	resection	yes
**Fambrini M. et al.**	2010	Case Report	1	45-year-old female	f	endometriosis	pelvic pain	no	yes	between adductor magnus and gracilis muscle	exopelvic	MRI	no	not further specified	Open surgical using an anterior approach from the thigh	resection	yes
**Kalkan Ü et al.**	2019	Case Report	1	25-year old female	f	deep infiltrating endometriosis	groin pain radiating towards inner thigh	yes	not further specified	retroperitoneally medial to external iliac vessels, lateral to utero-sacral ligament	endopelvic	MRI	no	not further specified	laparoscopy	resection	yes

**Langebrekke, A. and Qvigstad, E.**	2009	Case Report	1	26-year old female	f	endometriosis and fibrotic tissue	pain (not further specified), adductor weakness	yes	not further specified	right obturator fossa	endopelvic	MRI	no	not further specified	laparoscopy	resection	yes
**Osorio F. et al.**	2018	Case Report	1	32-year-old female	f	endometriosis	inner thigh pain	no	not further specified	obturator internus muscle below right iliac external vein	endopelvic	MRI	no	not further specified	laparoscopy	resection	yes
**Peters A. et al.**	2020	Case Report	1	32-year-old female	f	deep infiltrating endometriosis	not further specified	no	not further specified	retroperitoneally in pelvic side all near external iliac vein, internal iliac artery, medial umbilical ligament and ureter	endopelvic	not further specified	no	not further specified	laparoscopy	resection	yes
**Waer P et al**	2012	Case Report	1	30-year-old female	f	endometriosis	hip pain	no	yes	along whole nerve	endo- and exopelvic	MRI	yes	no	not further specified	resection	yes

Table S3: An overview of the studies included in the “endometriosis” group is presented. The location of obturator nerve irritation is illustrated in detail and categorized based on its relationship to the pelvis. (CT: computed tomography; MRI: magnetic resonance imaging; N: number of patients; f: female; m: male; n/a: not applicable)

Authors	Year	Study Design	N	Popula-tion	sex	De-scripted cause of nerve ir-ritation	leading symptom	motor weak-ness as leading symptom	de-scripted anatomic location of irrita-tion	location of irrita-tion ac-cording to the pelvis	Diagnostic modality	Biopsy (yes/ no)	Mononeuropathy on EMG	Treatment approach	Treatment type	Treat-ment success
**Kleiner, J. B. and Thorne, R. P.**	1989	Case Re-port	1	63-year old male	m	hypogas-tric artery aneurysm	buttock, groin and antero-medial thigh pain	no	Retroperi-toneally (not further specified)	endopel-vic	CT, arteri-ography, MRI	no	yes	laparot-omy	artery li-gation	yes
**Kubacz, G. J.**	1971	Case Re-port	1	79-year old-fe-male	f	ruptured aneurysm of ab-dominal aorta	Thigh and leg pain	yes	belly of the psoase muscle	endopel-vic	autopsy	no	n/a	n/a	n/a	no
**Lazaro et al.**	1981	Case Re-port	1	80-year-old male	m	retroperi-toneal hemor-rhage	antero-medial thigh sen-sory loss, obturator muscle weakness	yes	psoas fascia	endopel-vic	CT	no	yes	conserva-tive	conserva-tive	yes

Table S4: An overview of the studies included in the “vascular” group is presented. The location of obturator nerve irritation is illustrated in detail and categorized based on its relationship to the pelvis. (CT: computed tomography; MRI: magnetic resonance imaging; N: number of patients; f: female; m: male; n/a: not applicable)

Authors	Year	Study Design	N	Population	sex	Describted cause of nerve irritation	leading symptom	motor weakness as leading symptom	describted anatomic location of irritation	location of irritation according to the pelvis	Diagnostic modality	Biopsy (yes/ no)	Mononeuropathy on EMG	Treatment approach	Treatment type	Treatment success
**Bachar Avnieli I. et al.**	2018	Case Report	1	52-year-old male	m	ganglion cyst	inner thigh pain	no	intra-pelvic	endopelvic	MRI	no	n/a	arthroscopy	resection	yes
**Botchu R. et al**	2013	Case Reports	2	67-year-old female and 38-year old male	mixed	ganglion from transverse acetabular ligament	buttock and groin pain	no	obturator foramen	obturator foramen	MRI	no	n/a	ultrasound-guided aspiration	aspiration	yes
**Campeas S and Rafii M.**	2002	Case Report	1	33-year old male	m	ganglion cyst	inner thigh pain	no	obturator foramen	obturator foramen	MRI	no		surgical lateral incision	resection	yes
**de Bruijn, K. M., et al.**	2013	Case Report	1	70-year-old male	m	joint connected ganglionic cyst	groin pain radiating towards inner thigh	yes	obturator foramen	obturator foramen	MRI	no	n/a	CT-guided punctation	aspiration	yes
**Jafarnia J et al.**	2022	Case Report	1	68-year-old male	m	acetabular paralabral cyst	groin pain	no	proximal of obturator foramen	endopelvic	MRI	no	n/a	Open surgical using an anterior approach from the thigh	resection	yes
**Jitpun et al.**	2019	Retrospective Study	1	46-year old male	m	intraneural ganglion cyst	groin pain radiating towards anterior and inner thigh	yes	obturator foramen	obturator foramen	MRI	no	yes	Open surgical using an anterior approach from the thigh	labral tear reparation	yes
**Jitpun et al.**	2019	Retrospective Study	1	59-year old woman	f	intraneural ganglion cyst	groin pain radiating towards anterior and inner thigh	yes	course of obturator nerve from obturator canal up to pelvic brim	endopelvic	MRI	no	yes	Open surgical using an anterior approach from the thigh	resection, neurolysis	yes

**Kim et al.**	2014	Case Report	1	63-year-old-male	m	acetabular paralabral cyst	inner thigh pain	no	right anteriorinferior aspect of acetabulum extending to obturator muscle	exopelvic	MRI	no	yes	conservative	conservative	yes
**Munugani S. et al.**	2019	Case Report	1	59-year old female	f	ganglion cyst	groin pain radiating towards inner thigh	no	obturator foramen	obturator foramen	MRI	no	n/a	laparotomy	resection	yes
**Schwabegger AH, Shafighi M, Gurunluoglu R**	2004	Case Report	1	34-year-old sportsman	m	ganglion cyst	thigh adduction weakness and pelvic pain	yes	obturator foramen	obturator foramen	MRI	yes	yes	laparoscopy	resection	yes
**Stuplich M. et al**	2005	Case Report	1	57-year old male	m	synovial cyst	buttock and groin pain radiating downward to thigh and leg	no	external obturator muscle	exopelvic	MRI	no	yes	arthroscopy	emptying of cyst	yes
**Sureka J. et al.**	2012	Case Report	1	26-year-old male	m	gangion cyst intranerural cyst	thigh pain	yes	intra neural ganglion cyst	endo- and exopelvic	MRI	no	n/a	not further specified	not further specified	not further specified
**Uchida A. et al.**	2006	Case Report	1	71-year-old male	m	mucoid pseudocyst	leg pain (not further specified)	no	cyst of nerve; near urinary bladder, extending into obturator foramen	endopelvic	MRI	no	n/a	laparotomy	resection	yes
**Vidoni A. et al.**	2019	Case Report	1	35-year-olde male	m	ganglion cyst from transverse acetabular ligament	buttock and groin pain	no	between the adductor longus (AL) and adductor brevis (AB)	exopelvic	MRI	no	n/a	CT-guided punctation	aspiration	yes

**Yukata K. et al.**	2005	Case Report	1	75-year-old female	f	labral cyst	groin pain radiating towards anterior and inner thigh	no	lateral wall of lesser pelvis	endopelvic	MRI	no	n/a	ultrasound-guided	aspiration	yes

Table S5: An overview of the studies included in the “cyst” group is presented. The location of obturator nerve irritation is illustrated in detail and categorized based on its relationship to the pelvis. (CT: computed tomography; MRI: magnetic resonance imaging; N: number of patients; f: female; m: male; n/a: not applicable)

Authors	Year	Study Design	N	Population	sex	Descripted cause of nerve irritation	leading symptom	motor weakness as leading symptom	descripted anatomic location of irritation	location of irritation according to the pelvis	Diagnostic modality	Biopsy (yes/ no)	Mononeuropathy on EMG	Treatment approach	Treatment type	Treatment success
**Bradshaw, C. and McCrory, P.**	1997	Case Report	1	male footballplayer	m	idiopathic	exercise-induced groin pain radiating towards inner thigh	no	Thigh Region	exopelvic	nerve block	no	yes	Open surgical using an anterior approach from the thigh	nerve decompression	yes
**Bradshaw, C. et al.**	1997	Prospective observationsal study	32	athletes	mixed	idiopathic	exercise-induced groin pain radiating towards inner thigh	no	Thigh Region	exopelvic	nerve block	no	yes	Open surgical using an anterior approach from the thigh	nerve decompression	yes
**Rigaud J. et al.**	2007	Case Report	1	52-year-old female	f	idiopathic	groin pain radiating towards inner thigh	no	obturator canal	obturator foramen	nerve block	no	no	laparoscopy	neurolysis	yes
**Rigaud J. et al.**	2009	Retrospective observational study	4	Adult Population	mixed	idiopathic	groin pain radiating towards anterior and inner thigh	no	obturator foramen	obturator foramen	nerve block	no	no	laparoscopy	neurolysis	yes
**Rigaud J. et al.**	2008	Retrospective observational study	3	Adult Population	mixed	idiopathic	groin pain radiating towards anterior and inner thigh	no	obturator foramen	obturator foramen	nerve block	no	no	laparoscopy	neurolysis	yes
**Siwiński, D.**	2005	Retrospective Study	52	athletes	mixed	idiopathic	exercise-induced groin pain radiating towards inner thigh	yes	Thigh Region	exopelvic	nerve block	no	yes	Open surgical using an anterior approach from the thigh	neurolysis	yes (41/52)

Table S6: An overview of the studies included in the “idiopathic” group is presented. The location of obturator nerve irritation is illustrated in detail and categorized based on its relationship to the pelvis. For cases in which no diagnosis was performed, it indicates n/a. (CT: computed tomography; MRI: magnetic resonance imaging; N: number of patients; f: female; m: male; n/a: not applicable)
